# Two Patients with Leprosy and the Sudden Appearance of Inflammation in the Skin and New Sensory Loss

**DOI:** 10.1371/journal.pntd.0000425

**Published:** 2009-09-29

**Authors:** Carlos Franco-Paredes, Jesse T. Jacob, Barbara Stryjewska, Leo Yoder

**Affiliations:** 1 Department of Medicine, Division of Infectious Diseases, Emory University School of Medicine, Atlanta, Georgia, United States of America; 2 Hospital Infantil de Mexico, Federico Gomez, Mexico City, Mexico; 3 National Hansen's Disease Program, Baton Rouge, Louisiana, United States of America; New York University School of Medicine, United States of America

Leprosy is a chronic infection caused by *Mycobacterium leprae* that affects the peripheral nerves, skin, and potentially other organs [1]–[5]. Although the worldwide prevalence of leprosy has decreased in the era of multi-drug therapy (MDT), the global detection of new cases of leprosy remains a concern, with more than 250,000 new cases of leprosy reported in 2007 [3]. The precise mechanism of transmission of leprosy has not been conclusively defined; however, it is likely that this occurs through respiratory secretions by untreated borderline lepromatous and polar lepromatous cases [1],[5]. Target cells of infection are macrophages, histiocytes in the skin, and the nonmyelinating and myelinating Schwann cells in the peripheral nerve, leading to axonal dysfunction and demyelination [6]. Nerve injury plays a central role in the pathogenesis of leprosy, leading to functional impairment and deformity of hands and feet and the eyes [1],[6]. Leprosy is diagnosed by definite loss of sensation in a hypopigmented or reddish skin patch, a thickened peripheral nerve with loss of sensation and muscle weakness in the affected nerve, and presence of acid-fast bacilli on skin smear or biopsy [1],[4].

The immunological response to *M. leprae* mounted by the host will determine the different potential clinical states. The Ridley-Joplin system uses clinical and histopathological features and the bacteriologic index and includes the polar categories (lepromatous [LL] and tuberculoid [TT]) and the borderline states (borderline tuberculoid [BT], borderline borderline [BB], and borderline lepromatous [BL]) [1] (Table 1). In the polar tuberculoid category, a Th1 type cell–mediated immune response with a low bacterial load is seen. Lepromatous states are characterized by low cell-mediated immunity and a higher bacterial load [5] (Table 1). Clinically, patients with tuberculoid leprosy have a single or very few hypopigmented macules or plaques with a raised edge; they are dry, scaly, hairless, and have reduced sensation; and only a few peripheral nerves are commonly enlarged [1]. Lepromatous leprosy is characterized by widely and symmetrically distributed skin macules, nodules, erythematous papules, and diffuse skin infiltration; thickened peripheral nerves are more frequently identified. Borderline states represent a mixture of signs and symptoms of the polar categories [1].

**Table 1:** World Health Organization System and Ridley-Joplin Classification and Type of Reaction.

WHO	Paucibacillary		Multibacillary		
Ridley-Joplin^a^	TT	BT	BB	BL^b^	LL
**Type 1 Reaction**	No	Yes	Yes	Yes	No
**Type 2 Reaction**	No	No	No	Yes	Yes

WHO classification is used for operational purposes in the field and it is based on the number of skin lesions; Ridley-Joplin classification is an immunopathological and clinical classification.

Adapted from [5].

a Ridley-Joplin classification: tuberculoid (TT); borderline tuberculoid (BT); borderline borderline (BB); borderline lepromatous (BL); and lepromatous (LL).

b Patients with BL can develop both types of reactions.

## Description of Case A

**Figure 1:**
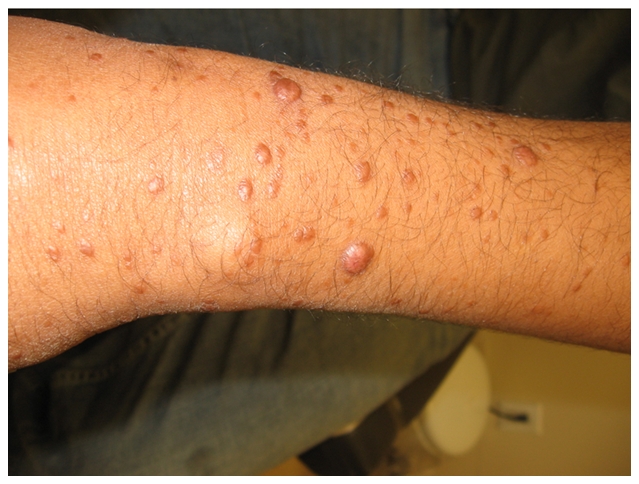
Multiple non-tender nodules (0.5–1.0 cm) in the right arm.

**Figure 2:**
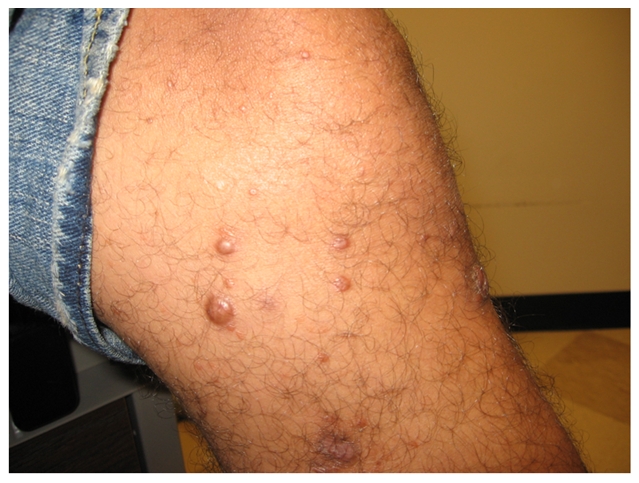
Multiple non-tender nodules (0.5–1.0 cm) in the right leg.

**Figure 3:**
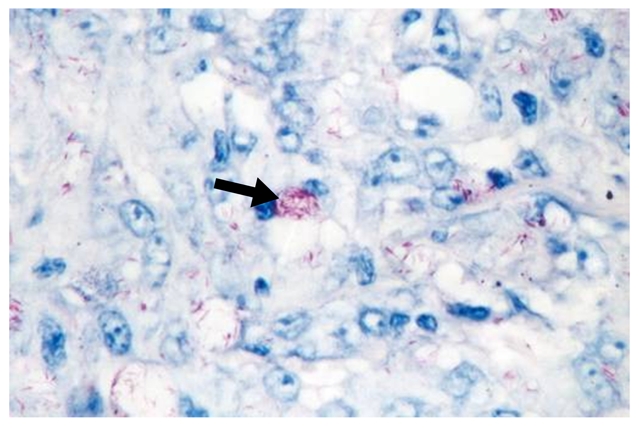
Fite-Faraco staining of skin biopsy demonstrating abundant acid-fast bacilli inside foamy macrophages (arrow).

A 31-year-old Brazilian male living in the United States for the previous four years presented with progressive crops of new nontender nodules on all four extremities over a 16-month period (Figures 1 and 2). He had more than ten nodules in each limb, with some reaching 0.5–1.0 cm in diameter. A 3×5-cm area of diffuse skin infiltration was present in his left thigh. He had referred numbness in the lower extremities. He had thickened bilateral ulnar nerves with mild sensory loss by monofilament testing in the ulnar nerve and peroneal nerve territories without any nerve tenderness identified. His posterior tibial nerves were also palpable. There was no muscle weakness identified by voluntary muscle testing using the 0–5 modified Medical Research Council scale. Nasal mucosa was normal. Eyelid closure was tested and there was no evidence of lagopthalmos; eyelashes were normal. His conjunctivae were pink. A skin biopsy demonstrated a diffuse lymphocytic infiltrate with multiple foamy macrophages. Fite-Faraco staining showed multiple acid-fast bacilli (Figure 3). A skin smear demonstrated a bacterial index (BI) of 5. The patient was diagnosed with lepromatous leprosy, using the Ridley-Joplin staging system [1], or multibacillary leprosy per the World Health Organization (WHO) staging [2]–[5] (Table 1). He was started on MDT consisting of dapsone 100 mg PO daily, rifampin 600 mg PO daily, and clofazimine 50 mg PO daily. (In the United States, the National Hansen's Disease Program recommends using daily rifampin, while the rest of the world uses rifampin once monthly with less than 1% relapses [4].)

We recommended that he continue his MDT until his BI (the BI is a logarithmic scale used to assess response to MDT in skin smears) decreased below 2 (dead bacteria may be present in the skin up to 10 years). Despite the WHO recommendation of 12 months of MDT for multibacillary cases such as our patient, many experts still advocate 24 months of MDT for patients with a BI >4 at diagnosis [1].

**Figure 4:**
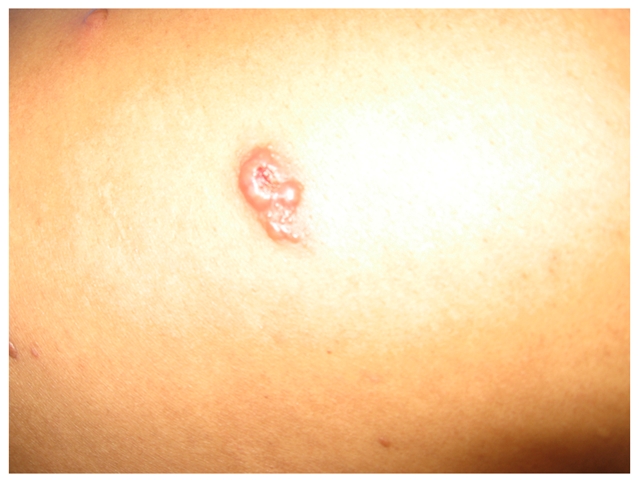
Newly appeared and rapidly ulcerating lesion in the right arm, accompanied with fever, cervical lymphadenopathy, and malaise.

Six months after the initiation of MDT, patient A experienced the abrupt onset of multiple new erythematous and ulcerated skin nodules (1–2 cm) distributed in the upper limbs, trunk, and right leg (Figure 4). The original leprosy nodules remained as they were. He also had a fever up to 39.5°C, swelling of his hands and feet, and cervical lymphadenopathy. He had thickened bilateral ulnar and peroneal nerves with moderate sensory loss by monofilament testing compared to his initial baseline evaluation in the ulnar nerve and peroneal nerve territories. There was no nerve tenderness identified. There was no muscle weakness identified by voluntary muscle testing. Histopathological examination of one new lesion showed evidence of neutrophilic infiltration. He had no clinical evidence of uveitis, arthritis, or orchitis.

Based on the clinical history and signs, our patient was diagnosed with a type 2 leprosy reaction [5] (Table 2). He was treated with 60 mg of oral prednisone daily tapered over a 12-week period [4]. A 6-week course of thalidomide 200 mg PO daily was also used due to the severity of the reaction, with rapid clinical improvement (Table 3). His rifampin dose was reduced to 600 mg monthly due to the concomitant use of prednisone since rifampin may lower prednisone serum levels. Our patient presented a protracted course with two more episodes of severe type 2 reactions that were treated with a similar corticosteroid regimen. A repeat skin biopsy performed 12 months after commencing MDT showed decreasing numbers of acid-fast bacilli, and skin smears showed a decreasing BI.

**Table 2:** Distinguishing Features of Type 1 and Type 2 Leprosy Reactions.

Leprosy Stage	Type 1 Reaction–Reversal Reaction (RR)	Type 2 Reaction–Erythema Nodosum Leprosum (ENL)
	Occurs in borderline disease (BT, BB, BL)^a^	Occurs in BL and LL^a^
**Skin**	Acute onset of redness and swelling in leprosy skin lesions and sometimes lesions may ulcerate. Marked edema of the hands, feet, and face may occur. No new lesions appear.	New painful and tender red papules or nodules which occur in crops in limbs or trunk and face. Ulceration of nodules may occur. Edema of the hands, feet, or face may occur. Original leprosy skin patches remain as they were.
**Nerve**	New nerve damage manifesting as numbness, or muscle weakness in the hands, feet, or face. Pain or tenderness in one or more nerves, with or without loss of nerve function.^b^	New nerve damage manifesting as numbness, or muscle weakness in the hands, feet, or face. Pain or tenderness in one or more nerves, with or without loss of nerve function.
**Systemic**	Unusual	Common, including fever, malaise; lymphadenitis, uveitis, neuritis, arthritis, dactylitis, orchitis.
**Diagnosis**	Clinical^c^	Clinical^d^
**Treatment**	Steroids^e^	Steroids

a BT  =  borderline tuberculoid; BB = borderline borderline; BL = borderline lepromatous; LL = lepromatous.

b Sometimes silent neuritis may be present, manifested by nerve function impairment (loss of sensations and/or muscle weakness) without skin inflammation.

c Skin biopsy may present some key features such as dermal edema, granuloma edema, and presence of giant cells and plasma cells.

d Skin biopsy may present a mixed dermal infiltrate of neutrophils and lymphocytes and fragmented bacilli in macrophages.

e For mild reactions, non-steroidal anti-inflammatory drugs may be sufficient. Prednisone or prednisolone are used for severe signs and symptoms; for alternative treatment options, see Table 2.

**Table 3:** Treatment of Type 1 and Type 2 Leprosy Reactions.

Type of Reaction	Manifestations/Treatment	Mild^a^	Severe^b^
**Type 1 Reaction (RR)**	**Clinical Manifestations**	• Erythematous, mildly swollen skin lesions; no painful or tender nerves	• Painful swollen skin lesions; swollen lesions of the face; edema of hands, feet, or face
		• No lesions of the face; no edema of the face, hands, or feet	• Diminished sensation or muscle weakness in hands and/or feet
	**Treatment**	• Non-steroidal analgesics (aspirin) for 1–2 weeks	• Prednisone^c^ or prednisolone^d^ 40–80 mg^e^ single daily dose then tapered over 12–20 weeks
			• In selected cases, clofazimine is added to prednisone as a steroid sparing agent in a dose up to 300 mg daily for 6 weeks. If effective, continue at reduced dose for additional 6–12 months
**Type 2 Reaction (ENL)** f	**Clinical Manifestations**	• No fever; minimal pain and no ulcerating lesions	• Febrile systemic illness; painful or ulcerating skin lesions; marked lymphadenitis; painful or tender nerves; diminished sensation or muscle weakness of hands or feet; severe edema of hands or feet; iritis, scleritis, arthritis, orchitis
		• No painful or tender nerves; no eye problems	
	**Treatment**	• Non-steroidal analgesics for 1–2 weeks	• Prednisone or prednisolone 40–80 mg daily tapered to lowest dose required to control the reaction
		• This regimen may be repeated several times if reaction remains mild	• Clofazimine may be used alone in patients intolerant to corticosteroids, or in combination with corticosteroids in those with severe type 2 reactions at a dose of 300 mg daily for 12 weeks (can be divided three times a day), and tapered to 100 mg twice a day for 12 weeks and then 100 mg once a day for 12–24 weeks
			• Thalidomide 200–400 mg daily in divided doses is sometimes used when available in combination with corticosteroids to control severe type 2 reactions^g^

Adapted from [5].

a Distinction between mild and severe reactions is clinical and based on the presence of one or more of the signs/symptoms listed.

b Use of steroids is recommended when patients have severe symptoms suggestive of new nerve damage (numbness and/or muscle weakness in the hands or feet). MDT should be continued concomitantly to anti-reaction drugs. In those who have completed MDT, management of reactions does not require restarting MDT [2].

c Patients receiving corticosteroids need to be examined every week and the dose of corticosteroid reduced every 2 weeks.

d Prednisone and prednisolone are equivalent in dosing potency and can be used interchangeably (see http://www.globalrph.com/corticocalc.htm). Some countries have availability of either prednisone or prednisolone [5].

e The WHO recommended regimen of corticosteroids (prednisone) is 40 mg daily for weeks 1 and 2; followed by 30 mg daily for weeks 3 and 4; 20 mg daily for weeks 5 and 6; 15 mg daily for weeks 7 and 8; 10 md daily for weeks 9 and 10, and 5 mg daily for weeks 11 and 12 [4].

f Severe type 2 reactions are often recurrent or chronic and therefore anti-reaction drug treatment may be adjusted according to the needs of the individual patient [11].

g WHO does not assist or support the use of thalidomide due to its teratogenic effects and its elevated cost. An additional argument is that most patients with type 2 reactions be can be successfully managed with the proper use of other anti-reaction drugs (corticosteroids and clofazimine) [4].

### What Are Leprosy Reactions and How Are They Diagnosed?

Reactional states represent a change in the immunological state of a patient with leprosy. These reactions are characterized by inflammatory episodes of the skin, nerves, and other organs that can occur anytime within the course of leprosy, but are often observed after starting treatment, and can result in irreversible nerve damage [2],[4]. There are two main types of reactions: type 1, or reversal reactions (RR), and type 2 reactions, or erythema nodosum leprosum (ENL) [5] (Table 2).

Type 1 reactions are considered to be an upgrading in the cell-mediated immune response to *M. leprae* [5]. These patients have relatively few bacteria and low antibody levels to *M. leprae* as compared to lepromatous states [1]. Schwann cells have been shown to express Toll-like receptor 2 (TLR-2) and major histocompatiblity complex (MHC II) with antigen presentation ability, resulting in CD4+ T cell activation [1],[7]. The incidence of type 1 reactions occurring as immune-reconstitution syndromes among patients with borderline leprosy co-infected with HIV/AIDS receiving antiretroviral therapy supports an upgrading of cell-mediated immunity as a key pathogenic event in type 1 reactions [8].

Type 2 reactions are characterized by an inflammatory infiltrate of neutrophils with associated vasculitis and/or panniculitis [9]. Although it is accepted that a cell-mediated component is required, the ultimate pathogenesis results from deposition of antigen–antibody complexes in tissues, leading to an acute inflammatory response, particularly in nerves, skin, and other organs with increased levels of circulating TNF-α and IL-6 [9]. The major T cell subtype identified during type 2 reactions is the CD4+ lymphocyte subset [9].

Type 1 reactions affect patients within the borderline spectrum (BT, BB, and BL), while type 2 reactions occur among patients with BL and LL states [5] (Tables 1 and 2). The higher the bacterial load and the greater the infiltration of the skin, the higher the risk of developing type 2 reactions [1],[8]. For type 1 reactions, starting MDT is considered the strongest risk factor [1].

In the MDT era, reactions occur in approximately 15%–39% of patients with leprosy [10]–[12].

### How Do Type 2 Reactions Present Clinically?

The diagnosis of leprosy reactions is based primarily on clinical history and examination [1],[5]. Type 2 reactions present with crops of new tender subcutaneous nodules sometimes associated with fever, malaise, arthralgias, neuritis, and adenopathy, and occasionally uveitis, orchitis, or dactylitis [1],[2],[5] (Table 2). Type 2 reactions usually occur after a patient with leprosy has been on MDT for months or years. Histologically, skin biopsy specimens may demonstrate a mixed dermal infiltrate of neutrophils and lymphocytes and fragmented bacilli in macrophages [2],[5],[13].

Type 2 reactions often follow a protracted clinical course [13]. In fact, three subtypes of type 2 reactions have been identified depending on its clinical course: acute single episode, multiple acute episodes, and chronic. Most cases tend to become chronic [13].

### What Is the Optimal Management of Type 2 Reactions?

MDT should be continued concomitantly to anti-reaction drugs in type 1 and type 2 reactions. In those who have completed MDT, management of reactions does not require restarting MDT [2]. For mild reactions, non-steroidal anti-inflammatory agents may be used (Table 3) [5]. Corticosteroids (prednisone or prednisolone) remain the standard of care for the acute symptoms of severe type 2 reactions with daily regimens tapered over a 12-week course [1],[2],[4],[5]. However, many patients require repeated treatment for multiple acute episodes or long-term corticosteroid use [2],[13].

Thalidomide is sometimes used in combination with corticosteroids for controlling severe type 2 reactions [10]. Its therapeutic effect has been associated with transient immune stimulation of IL-2 and interferon-γ produced by activated CD4+ and CD8+ lymphocytes [14]. Nonetheless, thalidomide has significant limitations for widespread use: it is costly, unavailable in many settings, and has significant side effects (teratogenicity, sedation, neuropathy, and thrombosis) [4].

Clofazimine is considered an anti-reaction agent in addition to its role in MDT against *M. leprae* (Table 3). Management of type 2 reactions with clofazimine is indicated in patients who are not responding satisfactorily to treatment with corticosteroids or when the risk of toxicity with corticosteroids is high [2]; or as monotherapy when corticosteroids are contraindicated [4]. The total duration of treatment with high dose clofazimine should not exceed 12 months. Side effects of clofazimine include gastrointestinal symptoms and skin hyperpigmentation [2],[4].

The use of tumor necrosis factor inhibitors (TNF-α inhibitors) has been considered in patients with type 2 reactions. However, the TNF-α inhibitor infliximab has been associated with the clinical development of leprosy and, upon its discontinuation, the occurrence of type 1 reactions [15]. The effects of other TNF inhibitors are not known. Azathioprine has been successfully used in preventing recurrences [16]. Mycophenolate mofetil is not effective when used as a corticosteroid sparing agent [17].

## Description of Case B

**Figure 5:**
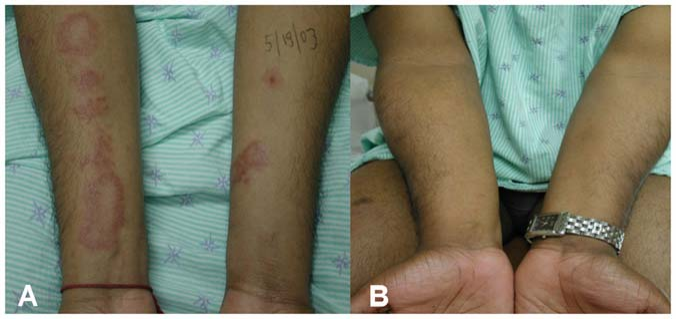
Patient with a type 1 leprosy reaction. (A) Patient with borderline tuberculoid leprosy with inflammation of previously existent skin plaques distributed in his four limbs and in the thorax. (B) Improvement of inflammation of the skin findings after corticosteroid treatment.

A 28-year-old Indian male, living in the United States for 2 years, presented with an 8-month history of multiple skin plaques that were well defined with their edges slightly raised, and scaly, and localized mostly in the face, upper and lower limbs, and trunk (Figure 5A). Some of these plaques were anesthetic and dry. The lesions range from 2 cm to 6 cm in diameter, with the ones on the right thigh being the largest. No thickened peripheral nerves or nerve tenderness were identified. Monofilament testing demonstrated mild sensory loss, more in the right hand than the left, and mainly in the distribution of the ulnar nerve. There was no other sensory loss detected at other sites and there was no muscle weakness identified by voluntary muscle testing using the 0–5 modified Medical Research Council scale. Nasal mucosa was normal. There was no evidence of madarosis with normal eyelashes, corneal reflexes, and eyelid closure; his conjunctivae were pink. Fite-Faraco stains of skin biopsy taken from one of the lesions in the right elbow demonstrated acid-fast organisms within histiocytes and within nerve fibers. A diagnosis of borderline tuberculoid was made (Tables 1 and 2) and he began receiving MDT consisting of rifampin 600 mg once a month, dapsone 100 mg daily, and clofazimine 50 mg daily.

Almost 2 months after he began receiving MDT, he developed a sudden onset of bilateral lower limb edema, with inflammation of previously existent skin plaques distributed in his four limbs and in the trunk. No new skin lesions were identified (Figure 5A). He also developed neuropathic pain in his lower limbs. No thickened peripheral nerves or nerve tenderness were identified. Monofilament testing demonstrated worsening sensory loss in the distribution of the ulnar nerves, and new mild sensory loss in the peroneal nerve distribution bilaterally. His voluntary muscle testing remained normal. At this point, our patient was diagnosed with a type 1 reaction. Along with continuing his MDT, he received a corticosteroid regimen tapered over a 20-week period, resulting in significant improvement (Figure 5B). He completed a 6-month course of MDT and has finished his prednisone tapered over a 9-month period.

### How Do Type 1 Reactions Present Clinically?

Type 1 reactions are characterized by increasing swelling, erythema, and tenderness of previously existing skin lesions accompanied by increased neuritis manifested as pain or tenderness in one or more nerves associated with pain and nerve function impairment [2],[5] (Table 2). Involvement of the face may result in facial paralysis and lagopthalmos; when affecting the eyes it may cause iritis and scleritis [5]. In type 1 reactions, some key features identified on skin biopsies include dermal edema, granuloma edema, and presence of giant cells and plasma cells [18].

### What Is the Optimal Management of a Type 1 Reaction?

The foremost priority in the management of type 1 reactions is the treatment of neuritis followed by decreasing acute inflammation, easing pain, and reversing nerve and eye damage [1],[5]. Corticosteroids (prednisone or prednisolone) are the standard of care [1], [2]–[5]. WHO recommends a regimen of corticosteroids tapered over a 12-week period (Table 3) [4]. However, regimens of corticosteroids tapered over a longer period (20 weeks) have been shown to be more effective in controlling type 1 reactions [1]. The most commonly affected nerves are the facial, ulnar, median, common peroneal, facial, and posterior tibial.

If the reversal reaction becomes chronic, requiring high doses of corticosteroids for extended periods, adjunctive clofazimine, started at 100 mg three times daily and subsequently tapered over many months, may be used (Table 3) [5]. Cyclosporine-A and methrotexate may be effective in patients intolerant to corticosteroids [19],[20].

## Discussion of Cases A and B

The clinical management of leprosy reactions is challenging. Leprosy reactions occur among patients with borderline and lepromatous leprosy and may occur prior to the initiation of MDT, during MDT, or even years after its completion. Corticosteroids are considered the standard of care for managing type 1 and type 2 reactions, sometimes in combination with clofazimine or thalidomide. Long-term follow-up of patients diagnosed with leprosy and who have suffered leprosy reactions is needed, even after completion of MDT. Further research regarding their pathogenesis and optimal medical management is warranted.

**Key Learning Points**

- Leprosy reactions contribute significantly to neurological sequelae and functional and anatomical impairment.
- Leprosy reactions occur among patients with borderline and lepromatous leprosy and may occur prior to the initiation of MDT, during MDT, or even years after its completion.
- Early detection and effective treatment of reactions is critical to prevent nerve damage.
- The treatment of choice for both types of reactions (and neuritis) is corticosteroids.

## Supporting Information

Alternative Language Abstract S1 Translation of the abstract into Spanish by Carlos Franco-Paredes
(0.03 MB DOC)
