# Supplementary material for: Two Patients with Leprosy and the Sudden Appearance of Inflammation in the Skin and New Sensory Loss
Source: PLoS Negl Trop Dis. 2009 Sep 29;3(9):e425. doi: 10.1371/journal.pntd.0000425 (PMC2742893; doi:10.1371/journal.pntd.0000425)
Supplement: Alternative Language Abstract S1 — Translation of the abstract into Spanish by Carlos Franco-Paredes (0.03 MB DOC) [file pntd.0000425.s001.doc]

| La lepra es una infección bacteriana crónica que afecta principalmente a los nervios periféricos y a la piel. Esta enfermedad infecciosa se puede presentar dentro de un amplio espectro bacteriológico, inmunológico, y clínico. Algunos pacientes con lepra pueden desarrollar complicaciones inflamatorias agudas denominadas “reacciones de la lepra”, las cuales pueden ocurrir antes de iniciar el tratamiento antimicobacteriano, durante el tratamiento, o posteriormente a completer el tratamiento. Estas reacciones son responsables en gran parte de las secuelas a largo plazo ocasionadas por la lepra y que incluyen deformaciones anatómicas, discapacidad y estigma asociado a la enfermedad. Existen dos tipos principales de reacciones de la lepra: tipo 1 (reacciones reversa) y tipo 2 (eritema nodoso leproso). Las reacciones se caracterizan por la aparicion súbita de eritema e inflamación de las lesiones en la piel. En ocasiones pueden aparecer nuevas lesiones en piel. Tambien puede haber inflamación y dolor en los nervios de los territorios afectados. La principal forma de tratamiento de las reacciones de la lepra son los glucocorticoides, los cuales reducen la inflamación en la piel y disminuyen el riesgo de discapacidad de los nervios periféricos ocasionados por la inflamación.  **Spanish translation by Carlos Franco-Paredes** |
| --- |
